# Kirkes' Hand-Book of Physiology

**Published:** 1900-12

**Authors:** 


**Kirkes' Hand-Book of Physiology.** By W. D. HALLIBURTON, M.D., F.R.S. Sixteenth Edition. Pp. xix., 872. London: John Murray. 1900.

It is always with pleasure that we read this book, perhaps from old associations, as it was our guide and friend when we first began to study physiology. It still keeps to the simple language, terseness of style, and homely analogies, which so well introduce the beginner to the scientific technicalities of language with which he must make himself familiar later on.

In this editon advantage has been taken of the opportunity to add matter here and there in order to bring the work fully up to date. There might perhaps be some changes effected in the diagrams of histology, such as a better one of hyaline cartilage, in which a *fibrous* matrix is clearly discernible, also in that supposed to represent Wharton's jelly, which, however, is very poorly represented in all the atlases which we have consulted; but these are merely quibbles, for the illustrations taken as a whole are most excellent, the diagrams being especially clear and thorough, as evidenced by the use of one (slightly modified) by Prof. Mott in his Croonian lectures.

We can cordially recommend the book to all students.

